# What We Observe Is Biased by What Other People Tell Us: Beliefs about the Reliability of Gaze Behavior Modulate Attentional Orienting to Gaze Cues

**DOI:** 10.1371/journal.pone.0094529

**Published:** 2014-04-10

**Authors:** Eva Wiese, Agnieszka Wykowska, Hermann J. Müller

**Affiliations:** 1 Department of General and Experimental Psychology, Ludwig-Maximilian-University, Munich, Germany; 2 Department of Psychology, George-Mason-University, Fairfax, Virginia, United States of America; 3 Institute for Cognitive Systems, Technical University, Munich, Germany; 4 Department of Psychological Sciences, Birkbeck College, University of London, London, United Kingdom; Centre de Neuroscience Cognitive, France

## Abstract

For effective social interactions with other people, information about the physical environment must be integrated with information about the interaction partner. In order to achieve this, processing of social information is guided by two components: a *bottom-up* mechanism reflexively triggered by stimulus-related information in the social scene and a *top-down* mechanism activated by task-related context information. In the present study, we investigated whether these components interact during attentional orienting to gaze direction. In particular, we examined whether the spatial specificity of gaze cueing is modulated by expectations about the reliability of gaze behavior. Expectations were either induced by instruction or could be derived from experience with displayed gaze behavior. Spatially specific cueing effects were observed with highly predictive gaze cues, but also when participants merely believed that actually non-predictive cues were highly predictive. Conversely, cueing effects for the whole gazed-at hemifield were observed with non-predictive gaze cues, and spatially specific cueing effects were attenuated when actually predictive gaze cues were believed to be non-predictive. This pattern indicates that (i) information about cue predictivity gained from sampling gaze behavior across social episodes can be incorporated in the attentional orienting to social cues, and that (ii) beliefs about gaze behavior modulate attentional orienting to gaze direction even when they contradict information available from social episodes.

## Introduction

In order to engage in interactions with other people we need to know *who* we are interacting with, and *what* others are going to do next [1]. Based on this knowledge, which can be acquired *directly* by interacting with people or *indirectly* by either observing someone interacting with another person or by receiving information about a person, we make inferences about the other's internal states, including intentions, beliefs, and feelings. The core of this *mentalizing* process [2] is that our predictions about others are based not simply on information about the state of the world, but also on our assumptions about the others' internal states. Accordingly, the interpretation of social scenes is thought to involve two components that interact with each other: (i) a *bottom-up* mechanism that is activated by perceptual information in the social scene, and (ii) a *top-down* mechanism that is based on background knowledge we have about others, or inferences we draw from perceived information. The combination of bottom-up and top-down processing ensures that our brain is able to react flexibly to the current situation while at the same time computes the most likely interpretation of the given perceptual input (based on context information about the interaction partner and the scene).

For understanding others in everyday situations, the human brain is equipped with a system that is specialized for processing social information, which consists of *medial prefrontal cortex* (mPFC), *superior temporal sulcus* (STS), *orbitofrontal cortex*, *amygdala* and *anterior insula* [3], [4]. Bottom-up responses to social signals are thought to be generated in the STS, which is particularly sensitive to biological movements (head/body movements, gestures, gaze direction/shifts) (for a review: [2]). Top-down modulation of these responses is assumed to originate from the mPFC (involved in mentalizing and processing of intentional behavior) and the amygdala (involved in the processing of the emotional content of the scene) [5], [6], [7], which help weight bottom-up signals according to their social relevance.

One of the most fundamental mechanisms employed in the processing of social information is following the gaze of others. Gaze direction is very informative, as it indicates their focus of interest and encourages the observer to shift attention to the same location (for a review: [8]). Gaze-triggered shifts of attention have been investigated using cueing paradigms [8], [9], in which a face is presented centrally that gazes either straight ahead, to the left, or to the right. Reactions to targets appearing in the gazed-at hemifield are typically faster than those to targets in the opposite hemifield [9], [10], [11].

Gaze direction has traditionally been thought to be special in guiding attention. In contrast to other central cues [12]–[14], gaze direction triggers shifts of attention to peripheral locations when it is not predictive [9], [10], [15] or even counter-predictive with respect to the target location [16] – a pattern that is consistent with a reflexive mechanism.

However, the view that gaze cues provide particularly powerful attentional orienting signals (reflecting their social relevance) has recently been challenged by evidence showing that not only gaze, but also other overlearned symbolic (e.g., arrow) cues are capable of inducing shifts of attention when they are not predictive [17]–[20], [16]. Furthermore, orienting attention in response to gaze direction can be top-down controlled if appropriate context information is available [10], [11], [21]. In particular, pre-existing assumptions concerning the observed stimulus have been shown to influence gaze cueing [22]–[26]: when humans believe that the observed gaze behavior is intentional, gaze cueing effects are larger compared to when the gazer is believed to display only mechanistic behavior [25], [27]. Similarly, when the gazer represents the leader of a group that the observer belongs to (e.g., a political party), the observer is more likely to follow his/her gaze direction [28].

Taken together, these findings suggest that gaze direction can evoke a top-down mechanism (in addition to a bottom-up mechanism that is always triggered), depending on whether or not task-relevant information is available. In support of this dual-component model, Wiese and colleagues [11] have shown that when targets were presented in an unstructured visual field, cueing was not specific to the exact gazed-at position, but facilitated all positions within the cued hemifield to an equal degree. However, when additional context information was provided in form of peripheral placeholders, cueing effects were the strongest for the exact gazed-at location. The authors took this pattern to indicate that bottom-up and top-down mechanisms are co-active in gaze following: while the bottom-up (*reflexive*) component causes a general directional bias for the whole cued hemifield, the top-down component triggers facilitation specific to the particular gazed-at position.

### Experiments

The present study was designed to investigate whether gaze-induced attentional orienting can be top-down modulated by the participants' expectations about the observed gaze behavior. Expectations were induced by either *actual predictivity* of gaze behavior (i.e., likelihood with which targets appeared at gazed-at locations) or *instructed predictivity* (independent of the actual predictivity). In Experiment 1, actual (i.e., experienced) predictivity tallied with instructed (i.e., believed) predictivity, so as to assess the combined influence of believed and experienced predictivity on the spatial specificity of gaze cueing. Experiment 2 examined whether an effect of cue predictivity on the spatial specificity of gaze cueing would also be observed when participants are not explicitly informed about the likelihood with which gaze cues indicate the target position (i.e., when instructions do not provide information about cue predictivity). Experiment 3 examined the spatial specificity of gaze cueing in conditions in which believed and experienced predictivity are in conflict (i.e., when high actual predictivity is believed to be low and low actual predictivity is believed to be high).

Based on the two-component model of Wiese et al. [11], we expected that when believed and actual predictivity are congruent, non-predictive displayed gaze behavior would activate the bottom-up component only, resulting in equal cueing effects for the whole hemifield. Predictive gaze behavior, by contrast, would additionally invoke the top-down component, giving rise to facilitation that is specific to the exact gazed-at position. Hence, in Experiment 1 (believed and actual predictivity congruent) we expected spatially specific cueing effects for highly predictive cues and non-specific cueing effects for non-predictive cues. If predictivity can be inferred from observing the gazer's behavior, then a similar pattern of effects should be observed in Experiment 2, where no explicit information about predictivity was given to participants. However, if observation-based inferences about cue predictivity are prone to influences from knowledge acquired through explicit instruction, the spatial specificity related to actual predictivity should be modulated by believed predictivity in Experiment 3. That is, nonspecific cueing effects triggered by non-predictive cues should become spatially more specific when the cue is believed to be predictive (Experiment 3), relative to when it is believed to be non-predictive (Experiment 1). By the same token, specific gaze-cueing effects induced by predictive cues should be less specific when the cue is believed to be non-predictive (Experiment 3) compared to when it is believed to be predictive (Experiment 1).

## Methods and Materials

### Experiment 1

**Figure 1:**
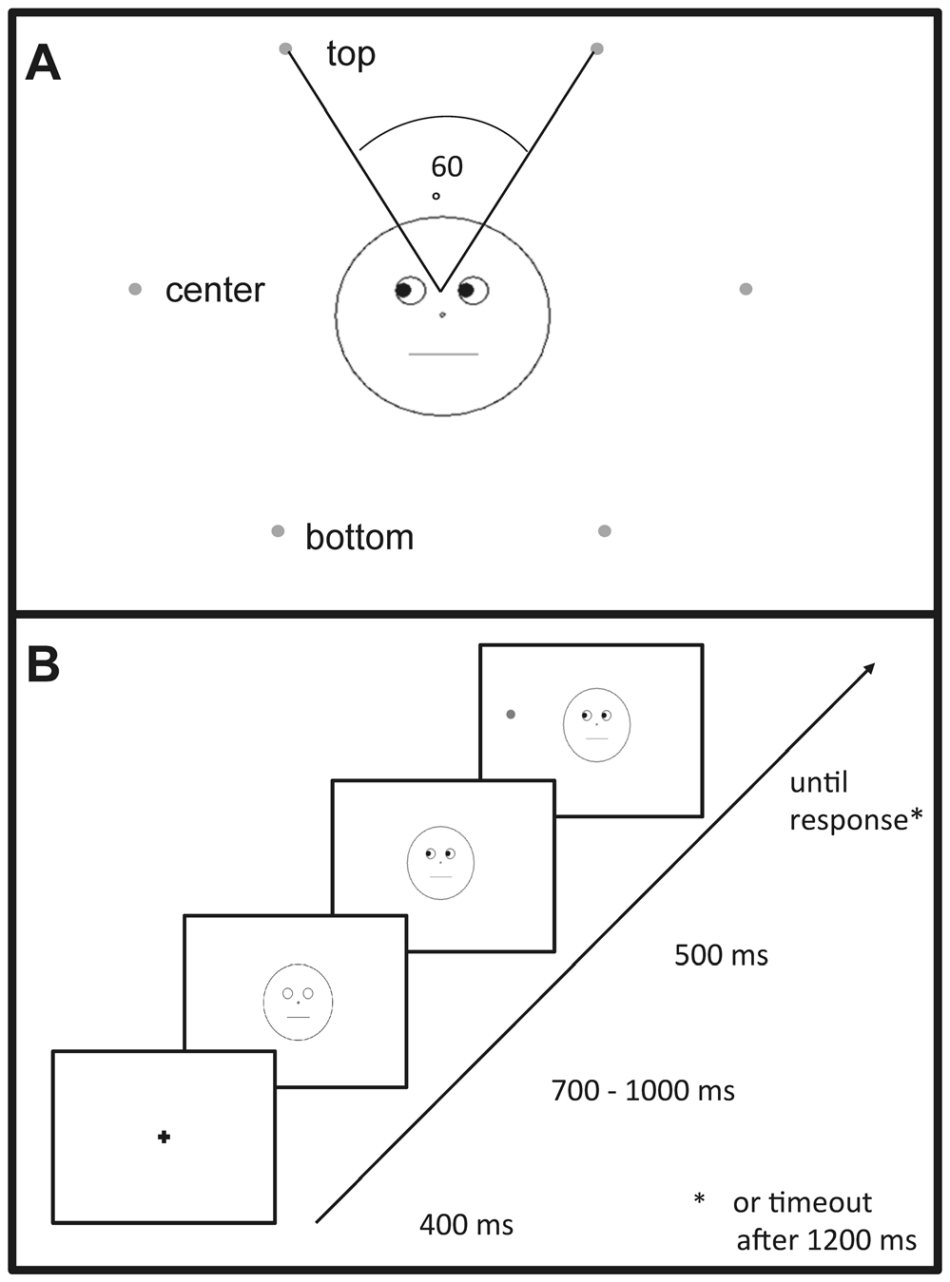
Stimulus and target positions (A) and sequence of events within a trial (B).

In Experiment 1, gaze cues either predicted the target location with a high likelihood (80%), or they were non-predictive (≈ 17%). Participants were explicitly informed about these probabilities. There were three semi-circularly arranged target positions in each hemifield, which were *not* marked by placeholders (See Figure 1A, and [11] for effects of non-predictive gaze cues without versus with placeholders). Participants had to make a speeded localization (left vs. right hemifield) response to the target. We expected predictive gaze cues to produce the strongest cueing effect for the exact gazed-at position, whereas non-predictive cues would generate equal cueing effects for all target positions within the cued hemifield.

#### Participants

Twelve volunteers (8 women; mean age: 25 years, range: 20–30 years; all right-handed, with normal or corrected-to-normal visual acuity) participated in the experiment either for course credit or payment (8€/h) and gave their written informed consent. The experimental procedure was approved by the ethics committee of the Department of Psychology, University of Munich, in accordance with the Code of Ethics of the World Medical Association (Declaration of Helsinki). Testing time was two hours, split into two sessions.

#### Apparatus

Stimuli were presented on a 17” Graphics Series G90fB CRT monitor with the refresh rate of 85 Hz. Reaction time (RT) measures were based on standard keyboard responses. Experiments were controlled by the software *Experiment Builder* (SR Research Ltd., Ontario, Canada). Participants were seated 57 cm away from the monitor, centered with respect to display and keyboard.

#### Stimuli

Schematic faces, constructed in line with Friesen and Kingstone [9], were presented in the center of the display as black drawings against a white background. The round face outline circumscribed an area of 6.8° of visual angle and contained two circles representing the eyes, a smaller circle symbolizing the nose, and a straight line representing the mouth. The eyes subtended 1.0° and were positioned on the horizontal midline, at a distance of ±1.0° from the vertical midline. The nose subtended 0.2°, was located 0.9° below the eyes, and served as fixation point. The mouth was 2.2° in length and centered 1.3° below the nose. Black filled circles, subtending 0.5°, appeared within the eyes, representing the pupils. Gaze cues were implemented by moving the pupils sideways into one of six different directions: pupils were either shifted left- or rightwards on the central horizontal axis or rotated up- or downwards relative to the midline by an angle of 60°, until they touched the outline eye circles. The target stimulus was a gray dot 0.5° in diameter. Targets could appear at one of six positions equally distributed on an imaginary circle with a radius of 6.0° around the fixation point within the central face (Figure 1A). The angular distance between adjacent targets was 60°.

#### Design

Each session of the experiment consisted of 740 trials, with a block of 20 practice trials preceding 20 experimental blocks of 36 trials each. Gaze direction (left, right), gaze position (top, center, bottom), target side (left, right), and target position (top, center, bottom) were presented pseudo-randomly. Cue predictivity was blocked: one testing session was devoted to non-predictive and the other to predictive cues, with session order counterbalanced across participants. In the non-predictive condition, targets appeared at each of the six target positions with the same likelihood (≈17%); by contrast, in the predictive condition, targets appeared with a likelihood of 80% at the exact gazed-at position and a likelihood of 4% each at one of the other five positions.

#### Procedure

Figure 1B illustrates the sequence of events on a trial. Trials started with the onset of a central fixation cross. 400 ms later, a face with blank eyes was presented. After a random interval of 700–1000 ms, pupils appeared within the eyes looking at one of the six target positions (Figure 1A). Following the cue, a target dot appeared at one of the six target positions at a stimulus onset asynchrony (SOA) of 500 ms. Schematic face, pupils, and target remained on the screen until a response was given or 1200 ms had elapsed. Participants were asked to determine, as fast and accurately as possible, whether targets were presented on the left or right side of the screen, pressing the “D”- or “K”-key with their left or right index finger for a target on the left or right side, respectively. The inter-trial-interval (ITI) was 680 ms.

Participants were veridically informed about the predictivity of the gaze cues: Instruction 1 stated that gaze direction was not predictive of the location of the upcoming target, and Instruction 2 informed them that the target would appear with a high likelihood at the gazed-at position.

#### Analysis

To examine whether the basic cueing effects were significant, the mean (correct) RTs were subjected to an ANOVA with the factors *validity* (valid, invalid), *gaze position* (top, center, bottom), *target position* (top, center, bottom), and *predictivity* (low, high).

The specificity of gaze cueing was assessed in a repeated-measures ANOVA on the gaze-cueing effects, with the factors *gaze position* (top, center, bottom), *target position* (top, center, bottom), and *predictivity* (low, high). Cueing effects were calculated as the RT-difference between a validly cued position (i.e., gaze direction and target side matched) and the respective invalidly cued position (i.e., gaze direction and target side did not match) on the same horizontal axis. For instance, cueing effects for the top-position (60° in the upper quadrant) on the left side were calculated as the RT-difference between trials on which this position was validly cued (i.e., gaze directed to the left) compared to when this position was invalidly cued (i.e., gaze directed to the right). For the ANOVA, cueing effects were collapsed across the two hemifields. Specific cueing effects would manifest as a significant interaction between gaze position and target position, with stronger cueing effects for the gazed-at position than for the other positions in the same hemifield. By contrast, non-specific gaze cueing would yield equal facilitation for all positions in the cued hemifield (i.e., a main effect of validity, in the absence of a gaze position x target position interaction on the cueing effects). If predictivity influenced the specificity of gaze cueing, the interaction among predictivity, gaze position, and target position should be significant, with the interaction between gaze and target position being significant only for predictive cues.

#### Results

**Figure 2:**
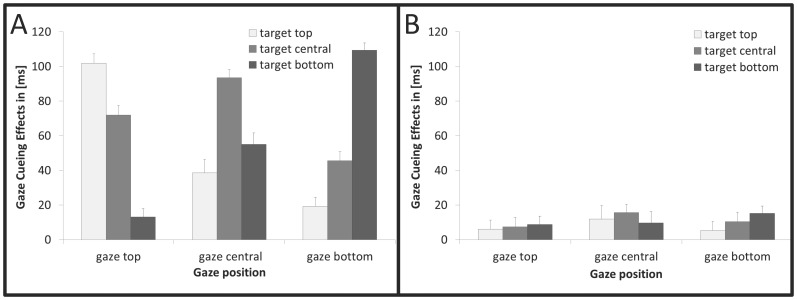
Gaze-cueing effects as function of gaze position and target position for (A) high actual and instructed predictivity; for (B) low actual and instructed predictivity. Depicted error bars represent standard errors of the mean adjusted to within-participants design.

Anticipations (defined as responses with latency <100 ms, 1.29%), misses (defined as responses with latency > 1200 ms, 3.69%), and incorrect responses (1.49%) were excluded from analysis. Please see Table S1 in Supplementary Materials for mean RTs and associated standard errors, and Table S2 for the results of the ANOVA on RTs. Results of follow-up ANOVAs on RTs, with the factors *validity* (valid, invalid), *gaze position* (top, center, bottom), *target position* (top, center, bottom), conducted separately for each predictivity condition are reported in Table S3. Figure 2 presents the cueing effects for predictive and non-predictive trials as a function of gaze position and target position. Results of the ANOVA on gaze-cueing effects are reported below.

The ANOVA of the RTs revealed a significant gaze cueing effect with shorter RTs for the valid compared to the invalid trials [validity: *F*(1,11) =  109.437, *p*<.001, η_P_^2^ = .909]. The ANOVA of the cueing effects revealed the gaze-cueing effects to be overall larger with predictive (Δ_RT_ =  61 ms) than with non-predictive cues (Δ_RT_ =  11 ms) [predictivity: *F*(1,11) =  44.716, *p*<.001, η_P_^2^ = .803]. Moreover, the spatial distribution of the gaze-cueing effects was dependent on the relation of the gazed position to the actual target position in the cued hemifield [gaze position x target position: *F*(4,44) =  18.716, *p*<.001, η_P_^2^ = .630]. Importantly, however, the spatial distribution of cueing effects differed significantly between predictive and non-predictive cues [predictivity x gaze position x target position: *F*(4,44) =  15.265, *p*<.001, η_P_^2^ = .581], with more specific cueing effects for the predictive compared to the non-predictive condition. All other effects were non-significant (all *F*s<2.543, all *p*s>.101, all η_P_^2^<.188).

To statistically test whether the spatially specific component manifested only with predictive, but not with non-predictive, cues, the cueing effects were examined in follow-up ANOVAs with only the factors *gaze position* (top, center, bottom) and *target position* (top, center, bottom), conducted separately for each of the predictivity conditions. With non-predictive cues, the cueing effects were of comparable size for all target positions in the cued hemifield [gaze position x target position: *F*(4,44) =  1.078, *p* = .379, η_P_^2^ = .088]; see Table S3 for the main effect of validity. By contrast, with predictive cues, the size of gaze-cueing effect depended on the congruency of the gazed-at and the target position [gaze position x target position: *F*(4,44) =  18.309, *p*<.001, η_P_^2^ = .625], with larger cueing effects for the gazed-at position compared to the other positions in the cued hemifield. All other effects were non-significant (all *F*s<1.973, all *p*s>.163, all η_P_^2^>.152).

To examine more directly whether cue predictivity had an influence on the spatial specificity of gaze cueing, we compared the size of cueing effects for the exact gazed-at position with the other two locations (averaged together) in the cued hemifield in a two-way ANOVA with the within-participants factors *location* (exact, other) and *predictivity* (high, low). Spatial specificity of gaze cueing was found to be strongly influenced by predictivity [*F*(1,11) =  31.461, *p*<.001, η_P_^2^ = .741] with significantly larger gaze-cueing effects for the exact gazed-at position than for the other two locations in the predictive condition (Δ_GCexact-other_ =  61 ms, *t*(11) =  6.111, *p*<.001, *d* = 1.89, *two-tailed*), but not in the non-predictive condition (Δ_GCexact-other_ =  3 ms, *t*(11) =  1.513, *p* = .159, *d* = .38, *two-tailed*). All T-tests were Bonferroni-corrected for multiple comparisons.

#### Discussion

Experiment 1 investigated whether attentional orienting to gaze direction is influenced by explicit (i.e., instructed) and implicit (i.e., experienced) information about the predictivity of gaze behavior. The results showed that for *predictive cues*, gaze cueing was significantly stronger for targets that appeared at the exact gazed-at position relative to targets that appeared at one of the other two positions in the cued hemifield. *Non-predictive cues*, by contrast, generated significant gaze-cueing effects (see Table S3) that were equally strong for all target positions within the cued hemifield.

The finding that predictivity influences both the size and spatial distribution of gaze-cueing effects raises an interesting question, namely: is the observed pattern mediated by instruction-induced expectations, or does it emerge as a result of acquired experience with gaze cues of various degrees of predictivity? The results of Experiment 1 alone cannot answer this question, as experienced ( = *actual*) and believed ( = *instructed*) predictivity were always congruent. The following two experiments were designed to disentangle the effects of experience versus belief. Experiment 2 investigated whether the pattern of results in Experiment 1 can be replicated when no explicit information is given about the cue predictivity (i.e., when no beliefs are induced), but when information about gaze–target contingencies can only be inferred from experience with the observed gaze behavior. In Experiment 3, we examined whether the spatial specificity that is induced by knowledge gained from experience with the actual cue predictivity (i.e., experienced predictivity) is modulated by knowledge acquired through instructions (i.e., believed predictivity) in conditions when these two sources of information are contrasted. To this end, believed and experienced predictivity were manipulated orthogonally in Experiment 3: in the high predictivity condition, participants were told that gaze cues are non-predictive; in the low predictivity condition, by contrast, participants were told that gaze cues are highly predictive.

### Experiment 2

In Experiment 2, we investigated the effect of experienced predictivity alone, that is: participants did not receive a-priori information about cue predictivity by instruction, but could deduce this information only from experience with displayed gaze behavior. If participants are able to deduce/learn predictivity through experience with the observed gaze behavior predictive gaze cues should produce the strongest cueing effect for the exact gazed-at position, whereas non-predictive cues should generate equal effects for all target positions within the cued hemifield, similar to Experiment 1.

Methodological details of Experiment 2 were the same as those of Experiment 1, with one exception: in Experiment 2, participants were not explicitly informed about cue predictivity in the instruction (i.e., no beliefs induced), so that they could infer this information only from their experience with the observed gaze behavior.

#### Participants

Twelve new volunteers (11 women; mean age: 25 years, range: 19–30 years; two left-handed, all with normal or corrected-to-normal visual acuity; all having given written informed consent) participated in Experiment 2, either for course credit or payment (8€/h).

#### Results and Discussion

Anticipations (1.79%), misses (0.08%), and incorrect responses (2.04%) were excluded from analysis. Table S4 in Supplementary Materials reports mean RTs and associated standard errors, and Table S5 shows the ANOVA results on RTs. ANOVA-results on gaze-cueing effects are summarized in Table S6, and effects of interest are reported below.

The ANOVA of the RTs revealed a significant gaze cueing effect with shorter RTs for the valid compared to the invalid conditions [validity: *F*(1,11) =  14.283, *p* = .003, η_P_^2^ = .192]. The ANOVA of the cueing effects revealed actual cue predictivity to influence the allocation of spatial attention induced by gaze cues: highly predictive cues gave rise to larger cueing effects (Δ_RT_ =  40 ms) than non-predictive cues (Δ_RT_ =  12 ms) [predictivity: *F*(1,11) =  10.765, *p* = .007, η_P_^2^ = .495]. Importantly, predictivity had a significant influence on the spatial specificity of gaze cueing, with general cueing effects for non-predictive cues and spatially specific cueing effects for the highly predictive cues [predictivity x gaze position x target position: *F*(4,44) =  5.018, *p* = .002, η_P_^2^ = .313].

To statistically test whether the spatially specific component manifested only with predictive, but not with non-predictive, cues, the cueing effects were examined in two follow-up ANOVAs (one for each predictivity condition) with the factors *gaze position* (top, center, bottom) and *target position* (top, center, bottom). With non-predictive cues, gaze cueing effects were of comparable size for all target positions in the cued hemifield [gaze position x target position: *F*(4,44) = .727, *p* = .578, η_P_^2^ = .062]. For predictive cues, by contrast, cueing effects were significantly larger at the gazed-at position compared to the other positions in the cued hemifield [gaze position x target position: *F*(4,44) =  5.229, *p* = .002, η_P_^2^ = .322].

The spatial specificity of gaze cueing was found to be strongly influenced by predictivity [*F*(1,11) =  15.989, *p* = .002, η_P_^2^ = .592], with significantly larger cueing effects for the exact gazed-at position than for the other two locations in the predictive condition (Δ_GCexact-other_ =  30 ms, *t*(11) =  3.982, *p* = .002, *d* =  1.05, *two-tailed*), but not in the non-predictive condition (Δ_GCexact-other_ =  3 ms, *t*(11) =  1.513, *p* = .159, *d* = .23, *two-tailed*). T-tests were Bonferroni-corrected for multiple comparisons.

### Experiment 3

In Experiment 3, the effects of actual and believed predictivity were contrasted. Participants received either *Instruction 1*: they were told that the cues were highly predictive, when they actually were non-predictive (*actual predictivity*: 17%; *instructed predictivity*: 80%); or *Instruction 2*: they were told that gaze cues were non-predictive, when they actually were highly predictive (*actual predictivity*: 80%, *instructed predictivity*: 17%). The order of instructions was counterbalanced across participants. To examine the influence of experienced versus believed predictivity on gaze cueing effects, we compared conditions with the same actual but different instructed predictivities. For that purpose, we conducted a four-way ANOVA of the gaze-cueing effects with the within-participant factors *gaze position* (top, center, bottom), *target position* (top, center, bottom), and *actual predictivity* (high, low), and the between-participant factor *experiment* (Experiment 1: experience congruent with instruction, Experiment 3: experience incongruent with instruction). In addition, we examined whether potential effects of believed predictivity on experienced predictivity changed over the course of the experiment, with a stronger influence of believed predictivity in the first half of the experiment and a stronger influence of experienced predictivity in the second half of the experiment. To this end, we conducted a four-way ANOVA of the gaze-cueing effects with the within-participant factors *gaze position* (top, center, bottom), *target position* (top, center, bottom), *predictivity* (high, low) and *half* (first, second).

Methods in Experiment 3 were similar to Experiment 1, with one exception: In Experiment 3, actual and instructed predictivity were incongruent, in contrast to Experiment 1 in which they were congruent.

#### Participants

Twelve new volunteers (10 women; mean age: 25 years, range: 20–28 years; all right-handed, all with normal or corrected-to-normal visual acuity; all having given written informed consent) participated in Experiment 3, either for course credit or payment (8€/h).

#### Results and Discussion

Anticipations (0.82%), misses (0.09%), and incorrect responses (3.86%) were excluded from analysis. Table S7 in Supplementary Materials reports mean RTs and associated standard errors, and Table S8 summarizes the ANOVA results on RTs. ANOVA-results on gaze-cueing effects are summarized in Table S9, and effects of interest are reported below.

**Figure 3:**
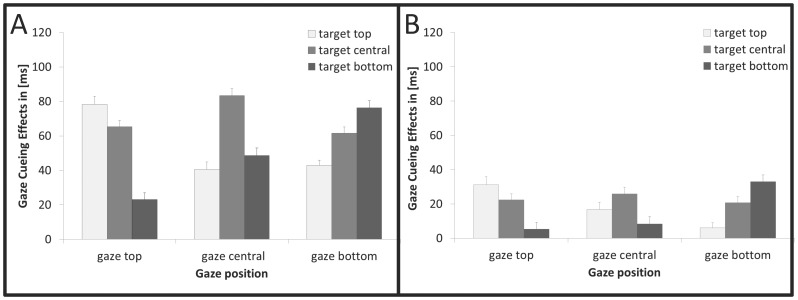
Gaze-cueing effects as function of gaze position and target position for (A) high actual predictivity and low instructed predictivity; for (B) low actual predictivity and high instructed predictivity. Depicted error bars represent corrected standard errors of the mean adjusted to within-participants design.

**Figure 4:**
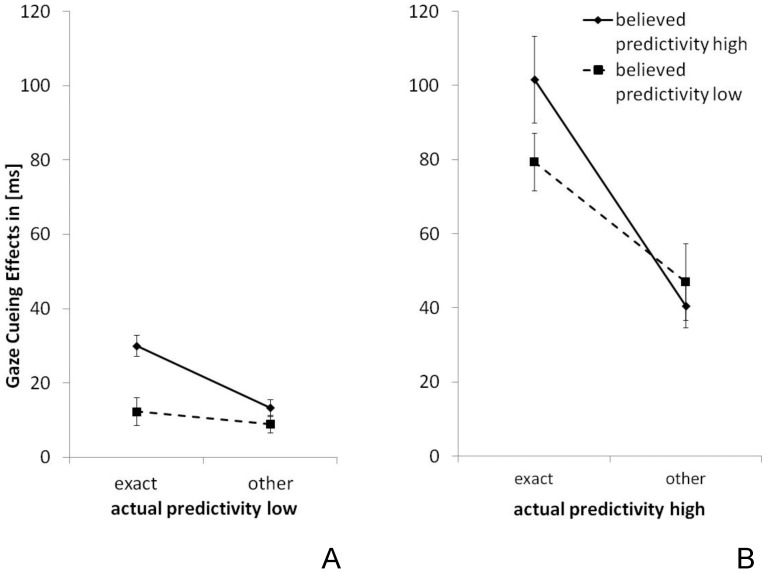
Comparison between Experiments. Gaze-cueing effects as function of target position (exact gazed-at position vs. other positions in cued hemifield), instructed predictivity (high: ***solid line***, low: ***dashed line***) and actual predictivity (high: ***left side***, low: ***right side***). Note that the bigger the difference (the steeper the depicted line) between gaze-cueing effects for the exact and the other positions in the cued hemifield, the more specific the allocation of attention to the gazed-at position. Depicted error bars represent corrected standard errors adjusted to within-subject designs.

The ANOVA of the RTs revealed a significant gaze cueing effect with shorter RTs for the valid compared to the invalid conditions [validity: *F*(1,11) =  59.829, *p*<.001, η_P_^2^ = .845]. The ANOVA of the cueing effects revealed actual cue predictivity to influence the allocation of spatial attention induced by gaze cues (see Figure 3): gaze cues with high actual predictivity gave rise to larger cueing effects than non-predictive cues [actual predictivity: *F*(1,22) =  64.975, *p*<.001, η_P_^2^ = .803]. Moreover, highly predictive cues generated cueing effects specific to the gazed-at position [actual predictivity x gaze position x target position: *F*(4,88) =  15.130, *p*<.001, η_P_^2^ = .407], with significant differences between the exact cued versus the other positions: all *t*s> 2.295, *p*s<.031, *d* >1.18, *two-tailed*). Crucially, this pattern was modulated by believed predictivity [experiment x actual predictivity x gaze position x target position: *F*(4,88) =  5.419, *p* = .001, η_P_^2^ = .198], that is: the allocation of spatial attention in response to the experienced (i.e., actual) cue predictivity was top-down modulated by expectations based on the believed (i.e., instructed) cue predictivity – see Figure 4.

In subsequent analyses, the spatial specificity of gaze cueing and its modulation by instructed predictivity was examined for high versus low predictivity conditions separately. *Non-predictive cues* generated nonspecific cueing effects when participants believed that the cue was not predictive (Exp.1), whereas the same cues produced specific effects when participants believed that the gaze cues were predictive (Exp.3) [experiment x gaze position x target position: *F*(4,88) =  5.649, *p*<.001, η_P_^2^ = .204]. Planned comparisons revealed significantly larger gaze-cueing effects for the exact gazed-at position than for the other positions within the cued hemifield when participants were told that the cues were predictive (Exp.3, Δ_GCcued-other_ =  17 ms), compared to when they were informed that the cues were non-predictive (Exp.1, Δ_GCcued-other_ =  3 ms); [*t*(21) =  3.478, *p* = .002, *d* = 1.42, two-tailed], see Figure 4A. Similarly, believed predictivity modulated the spatial specificity of gaze cueing for *predictive cues* [experiment x gaze position x target position: *F*(4,88) =  2.583, *p* = .043, η_P_^2^ = .105]: the spatially specific component was significantly stronger for cues believed to be predictive (Exp.1, Δ_GCcued-other_ =  61 ms) compared to cues believed to be non-predictive (Exp.3, Δ_GCcued-other_ =  32 ms), [*t(*21) =  −2.216, *p* = .037, *d* = 0.90, *two-tailed*], see Figure 4B. Complete results are reported in Table S10. All T-tests were Bonferroni-corrected for multiple comparisons.

Finally, we examined whether the interactive effect of believed and experienced predictivity on the specificity of gaze cueing changed over the course of the experiment, with a stronger effect of believed predictivity in the first half and a stronger influence of experienced predictivity in the second half of the experiment. We found no effect of *half* (first, second) on the spatial distribution of the gaze cueing effects [half x predictivity x gaze position x target position: *F*(4,44) =  1.761, *p* = .154, η_P_^2^ = .138], indicating that the top-down modulation of believed predictivity on experienced predictivity was stable throughout the experiment.

## General Discussion

The goal of the present study was to investigate whether fundamental mechanisms of social cognition such as orienting of attention in response to gaze direction are influenced by context information about the predictivity of observed gaze behavior. In three experiments, information about predictivity could be implicitly inferred from observed gaze behavior (i.e., experienced predictivity). In Experiment 1 and 3 (but not in Experiment 2), information about predictivity was also provided explicitly by instruction (i.e., believed predictivity): in these experiments, experienced predictivity either was (Experiment 1) or was not congruent (Experiment 3) with believed predictivity.

When actual and instructed predictivity matched (Experiment 1), we expected specific cueing effects for the exact gazed-at location in the predictive condition and cueing effects for the whole cued hemifield in the non-predictive condition. When no information about cue predictivity was given by instruction (Experiment 2), we expected specific cueing effects for high predictivity and nonspecific cueing effects for low predictivity, if participants were able to acquire information about gaze–target contingencies based on experience (similar to Experiment 1). Experiment 3 was designed to examine whether knowledge about cue predictivity gained through experience (i.e., experienced predictivity) interacts with knowledge acquired through instruction (i.e., believed predictivity). To this end, actual and instructed predictivity were made to mismatch in Experiment 3. On the assumption that knowledge about predictivity acquired through instruction interacts with knowledge about predictivity gained from experience, we expected that gaze cueing effects induced by highly predictive cues should be spatially less specific when they were believed to be non-predictive. By the same logic, cueing effects induced by non-predictive cues should become spatially more specific when they were believed to be highly predictive as to the target position.

Spatially specific cueing effects for highly predictive cues and non-specific cueing effects for non-predictive cues were predicted based on Wiese and colleagues [11], who showed that a general gaze-cueing effect for the whole gazed-at hemifield could be complemented by a cueing effect specific for the gazed-at position, when context information was provided in the scene (i.e., when peripheral position placeholders were presented that could be referred to by gaze). This pattern led the authors to propose a two-component model of gaze cueing, according to which specific gaze-cueing effects are mediated by a context-dependent top-down component that is integrated with a bottom-up component producing a general directional bias towards the gaze-cued hemifield.

The present findings provide further support for the two-component model. In the present study, gaze cueing was not modulated by visual context information (i.e., placeholders) but by believed and / or experienced context information about the reliability of gaze behavior: with predictive cues, gaze-cueing effects were significantly larger for targets that appeared at the exact gazed-at position relative to targets at the other two positions within the cued hemifield; non-predictive cues, by contrast, gave rise to cueing effects of equivalent magnitude for all positions within the cued hemifield. Importantly, the effects of experienced predictivity were modulated by expected predictivity: non-predictive cues believed to be predictive caused cueing effects specific to the gazed-at position, compared to non-predictive cues that were veridically instructed to be non-predictive (Figure 4A). In contrast, specific cueing effects caused by actually predictive cues were significantly reduced when the cue was believed to be non-predictive (Figure 4B).

The present results extend previous findings of Wiese and colleagues [11] by showing that gaze cueing effects may not only be up-, but also down-regulated depending on the context information that is provided about cue predictivity: a specific cueing effect caused by actually predictive cues is reduced in its spatial specificity when participants believe that the cue is non-predictive; by the same token, spatially non-specific cueing effects induced by actually non-predictive cues yield increased spatial specificity when participants are told that the cue is predictive (Figures 2, 3, and 4). Thus, together with previous findings, this study supports the view that top-down modulation of the spatial distribution of cueing effects can be induced by various types of context information: visual information provided in the scene (i.e., position placeholder), empirical knowledge (i.e., gained through experience), and verbal information (i.e., instruction about the reliability of gaze behavior).

Nevertheless, although the present results provide evidence for a modulation of gaze cueing effects by context information, it is less clear whether orienting to gaze in conditions without context information reflects a pure bottom-up mechanism. In this regard, one potential limitation of the present study is owing to the fact that an intermediate cue–target SOA (of 500 ms) was used in all experiments, while pure bottom-up effects are more likely observed at short SOAs. However, based on findings from classical gaze-cueing experiments [8], [9], there is no reason to assume that bottom-up effects cannot be found at longer SOAs. In fact, Friesen and Kingstone [9] have shown that when non-predictive gaze cues are used and no context information is given that would allow for top-down modulation, gaze-cueing effects are found for a broad range of SOAs (100, 300, 600, and 1000 ms). An even more striking demonstration of bottom-up orienting to gaze direction at long SOAs can be found in Friesen, Ristic, and Kingstone [29], who observed reflexive orienting to counter-predictive gaze cues at SOAs of 600 ms (compared to SOAs of 1200 or 1800 ms, at which participants voluntarily shifted attention to predicted locations). That is, SOA alone does not determine whether bottom-up and / or top-down processes are involved in attentional orienting to gaze direction; instead, the decisive factor is the availability of context information (e.g., about cue predictivity) that permits the observer to interpret gaze behavior in a socially meaningful way. Our study supports this interpretation by showing that although significant cueing effects were found in all conditions (even when actual and believed predictivity were low and no context information was provided) for an SOA of 500 ms, the size and spatial specificity of these cueing effects were modulated only if context information about the reliability of the cue was available.

The observation that explicit knowledge about *who* we are interacting with does influence basic attentional processes involved in social interactions is consistent with [1], [24], [25], [27], where it has been suggested that bottom-up orienting to gaze cues can be top-down controlled by contextual information about the gazer. Similarly, familiarity with the gazer (stimuli depicting participants' colleagues; gender effect for women: [22]) or belonging to the same group as the gazer (e.g., political party: [28]) has also been shown to modulate the size of gaze-cueing effects. Note, however, that these studies have demonstrated a modulation of gaze cueing only under very specific conditions, namely: when context information is pre-existing and not acquired during the experiment.

In contrast to previous studies, the present study shows that gaze cueing effects can also be modulated, when context information has to be acquired through experience. In particular, we showed that knowledge about gaze–target contingencies can be learned over the course of the experiment, which then modulates the size and the spatial specificity of the gaze-cueing effects: when the gazing face indicates target position with a high reliability, cueing effects are larger and spatially more specific than when gaze cues are not predictive of target location. This finding appears to be at variance with a previous study by Bayliss and Tipper [26], who found effects of predictivity on subjective judgments about the gazers' trustworthiness, but no modulation of gaze cueing effects when knowledge about the reliability of the gazer had to be inferred from experience. However, there is a substantial difference between Bayliss and Tipper's study [26] and the present experiments: in [26], information about the reliability of the gazer was coupled with facial identity (i.e., multiple different faces indicated target position with different likelihoods) and randomized throughout the experiment, whereas in the present study the same face was used throughout the whole experiment and information about predictivity was blocked. One problem arising from coupling gaze direction and facial identity in one experiment is that the interpretation of these two signals is subserved by different neural networks and that their outputs are integrated only at later stages of information processing [30]. Given that gaze cueing produces fast-acting effects on attentional orienting, it is likely that cueing studies fail to disclose effects of slower-acting facial identity information on the response to gaze cues.

In summary, our findings show that early operations of spatial attention are highly penetrable by cognitive processes related to social context. The involvement of a context-modulated mechanism in gaze cueing is very plausible, as gaze-triggered mechanisms of attention are specifically sensitive to the social relevance of the environment within which they operate: the bottom-up component assures a general preparedness to social signals conveyed by other people, while the top-down mechanism allows flexible adaptation to the social context of a scene. The present study shows that in integrating context information within social attention mechanisms, humans tend to incorporate what they are told about others into their own experience and observation.

## Supporting Information

Table S1 **Mean Response Times and Standard Errors (in ms) for actual and instructed predictivity low vs. high (** ***Exp. 1*** **).**
(DOC)

Table S2 **F- and p-values for the four-way ANOVA on RTs with the factors (i) validity, (ii) gaze position, (iii) target position, and (iv) predictivity (actual and instructed predictivity congruent,** ***Exp. 1*** **).**
(DOC)

Table S3 **F-values and p-values for the post-hoc (three-way) ANOVAs on RTs with the factors (i) validity, (ii) gaze position, and (iii) target position, conducted separately for each actual predictivity condition (** ***Exp. 1*** **).**
(DOC)

Table S4 **Mean Response Times and Standard Errors (in ms) for actual predictivity low vs. high (** ***Exp. 2*** **).**
(DOC)

Table S5 **F-values and p-values for the four-way ANOVA on RTs with the factors (i) validity, (ii) gaze position, (iii) target position, and (iv) actual predictivity (** ***Exp. 2*** **).**
(DOC)

Table S6 **F-values and p-values for the three-way ANOVA on gaze-cueing effects with the factors (i) gaze position, (ii) target position, and (iii) actual predictivity (** ***Exp. 2*** **).**
(DOC)

Table S7 **Mean Response Times and Standard Errors (in ms) for actual predictivity low/believed predictivity high vs. actual predictivity high/believed predictivity low (Exp.3).**
(DOC)

Table S8 **F-values and p-values for the four-way ANOVA on RTs with the factors (i) validity, (ii) gaze position, (iii) target position, and (iv) actual predictivity.**
(DOC)

Table S9 **F-values and p-values for the four-way ANOVA on gaze-cueing effects with the factors (i) gaze position, (ii) target position, (iii) actual predictivity, and (iv) experiment (instructed predictivity,** ***Exp. 3*** **).**
(DOC)

Table S10 **F-values and p-values for the three-way ANOVA on gaze-cueing effects with the factors (i) gaze position, (ii) target position, and (iii) experiment (instructed predictivity,** ***Exp. 3*** **) for actual predictivity high vs. low.**
(DOC)
