# Supplementary material for: What We Observe Is Biased by What Other People Tell Us: Beliefs about the Reliability of Gaze Behavior Modulate Attentional Orienting to Gaze Cues
Source: PLoS One. 2014 Apr 10;9(4):e94529. doi: 10.1371/journal.pone.0094529 (PMC3983279; doi:10.1371/journal.pone.0094529)
Supplement: Table S1 — Mean Response Times and Standard Errors (in ms) for actual and instructed predictivity low vs. high ( Exp. 1 ). (DOC) [file pone.0094529.s001.doc]

**Table S1.** Mean Response Times and Standard Errors (in ms) for **actual and instructed** predictivity high vs. low (*Exp. 1*).

|  |  | actual and instructed predictivity high | | |  | actual and instructed predictivity low | | |
| --- | --- | --- | --- | --- | --- | --- | --- | --- |
|  |  | Gaze top | Gaze central | Gaze bottom |  | Gaze top | Gaze central | Gaze bottom |
|  |  |  |  |  |  |  |  |  |
| Target top | valid | 279 (10) | 339 (24) | 378 (16) |  | 327 (13) | 328 (14) | 335 (13) |
|  | invalid | 381 (10) | 378 (21) | 398 (10) |  | 333 (12) | 341 (12) | 341 (12) |
| Target central | valid | 306 (11) | 261 (15) | 320 (13) |  | 310 (12) | 304 (10) | 312 (12) |
|  | invalid | 378 (12) | 354 (17) | 365 (16) |  | 318 (11) | 319 (10) | 323 (11) |
| Target bottom | valid | 355 (12) | 325 (24) | 273 (10) |  | 324 (12) | 323 (12) | 314 (13) |
|  | invalid | 368 (19) | 380 (21) | 382 (10) |  | 333 (12) | 333 (12) | 328 (12) |
