# Supplementary material for: What We Observe Is Biased by What Other People Tell Us: Beliefs about the Reliability of Gaze Behavior Modulate Attentional Orienting to Gaze Cues
Source: PLoS One. 2014 Apr 10;9(4):e94529. doi: 10.1371/journal.pone.0094529 (PMC3983279; doi:10.1371/journal.pone.0094529)
Supplement: Table S2 — F- and p-values for the four-way ANOVA on RTs with the factors (i) validity, (ii) gaze position, (iii) target position, and (iv) predictivity (actual and instructed predictivity congruent, Exp. 1 ). (DOC) [file pone.0094529.s002.doc]

**Table S2.**  F-and p-values for the four-way ANOVA on **RTs** with the factors: validity, gaze position, target position and predictivity (actual and instructed predictivity congruent, *Exp.1*).

|  |  |  | *F-*value | *p-*value | effect size |
| --- | --- | --- | --- | --- | --- |
|  |  |  |  |  |  |
| validity |  |  | *F*(1,11)= 109.437 | *p*< .001 | η_P_^2^= .909 |
| target position |  |  | *F*(2,22)= 49.954 | *p*< .001 | η_P_^2^= .820 |
| gaze position |  |  | *F*(2,22)= 1.090 | *p*= .354 | η_P_^2^= .090 |
| predictivity |  |  | *F*(1,11)= 1.057 | *p*= .326 | η_P_^2^= .088 |
| validity x target position |  |  | *F*(2,22)= 2.543 | *p=* .101 | η_P_^2^= .188 |
| validity x gaze position |  |  | *F*(2,22)= .526 | *p*= .598 | η_P_^2^= .046 |
| validity x predictivity |  |  | *F*(1,11)= 44.716 | *p*< .001 | η_P_^2^= .803 |
| gaze position x target position |  |  | *F*(4,44)= 14.766 | *p*< .001 | η_P_^2^= .573 |
| gaze position x predictivity |  |  | *F*(2,22)= .778 | *p*= .472 | η_P_^2^= .066 |
| target position x predictivity |  |  | *F*(2,22)= 1.247 | *p*= .307 | η_P_^2^= .102 |
| validity x gaze pos x target pos |  |  | *F*(4,44)= 18.716 | *p*< .001 | η_P_^2^= .630 |
| validity x gaze pos x predictivity |  |  | *F*(2,22)= .647 | *p*= .533 | η_P_^2^= .056 |
| validity x target pos x predictivity |  |  | *F*(2,22)= 1.268 | *p*= .301 | η_P_^2^= .103 |
| gaze pos x target pos x predictivity |  |  | *F*(4,44)= 8.560 | *p*< .001 | η_P_^2^= .438 |
| validity x gaze pos x target pos x predictivity |  |  | *F*(4,44)= 15.265 | *p*< .001 | η_P_^2^= .581 |
|  |  |  |  |  |  |
