# Supplementary material for: What We Observe Is Biased by What Other People Tell Us: Beliefs about the Reliability of Gaze Behavior Modulate Attentional Orienting to Gaze Cues
Source: PLoS One. 2014 Apr 10;9(4):e94529. doi: 10.1371/journal.pone.0094529 (PMC3983279; doi:10.1371/journal.pone.0094529)
Supplement: Table S3 — F-values and p-values for the post-hoc (three-way) ANOVAs on RTs with the factors (i) validity, (ii) gaze position, and (iii) target position, conducted separately for each actual predictivity condition ( Exp. 1 ). (DOC) [file pone.0094529.s003.doc]

Table S3. F-values and p-values for the post-hoc ANOVAs on RTs with the factors: validity, gaze position, and target position for each actual predictivity condition separately (*Exp.1*).

|  |  | actual and instructed predictivity high | | |  | actual and instructed predictivity low | | |
| --- | --- | --- | --- | --- | --- | --- | --- | --- |
|  |  | *F-*value | *p-*value | effect size |  | *F-*value | *p-*value | effect size |
|  |  |  |  |  |  |  |  |  |
| validity |  | *F*(1,11)= 79.447 | *p<* .001 | η_P_^2^= .878 |  | *F*(1,11)= 19.413 | *p=* .001 | η_P_^2^= .638 |
| target position |  | *F*(2,22)= 20.016 | *p<* .001 | η_P_^2^= .645 |  | *F*(2,22)= 32.301 | *p<* .001 | η_P_^2^= .746 |
| gaze position |  | *F*(2,22)= .948 | *p=* .403 | η_P_^2^= .079 |  | *F*(2,22)= .518 | *p=* .603 | η_P_^2^= .045 |
|  |  |  |  |  |  |  |  |  |
| validity x target position |  | *F*(2,22)= 1.973 | *p=* .163 | η_P_^2^= .152 |  | *F*(2,22)= 1.126 | *p=* .342 | η_P_^2^= .093 |
| validity x gaze position |  | *F*(2,22)= .346 | *p=* .711 | η_P_^2^= .031 |  | *F*(2,22)= 1.864 | *p=* .179 | η_P_^2^= .145 |
| target position x gaze position |  | *F*(4,44)= 12.276 | *p*< .001 | η_P_^2^= .527 |  | *F*(4,44)= 5.470 | *p=* .001 | η_P_^2^= .332 |
|  |  |  |  |  |  |  |  |  |
| validity x gaze pos x target pos |  | *F*(4,44)= 18.309 | *p<* .001 | η_P_^2^= .625 |  | *F*(4,44)= 1.078 | *p=* .379 | η_P_^2^= .089 |
|  |  |  |  |  |  |  |  |  |
