# Supplementary material for: What We Observe Is Biased by What Other People Tell Us: Beliefs about the Reliability of Gaze Behavior Modulate Attentional Orienting to Gaze Cues
Source: PLoS One. 2014 Apr 10;9(4):e94529. doi: 10.1371/journal.pone.0094529 (PMC3983279; doi:10.1371/journal.pone.0094529)
Supplement: Table S4 — Mean Response Times and Standard Errors (in ms) for actual predictivity low vs. high ( Exp. 2 ). (DOC) [file pone.0094529.s004.doc]

**Table S4.** Mean Response Times and Standard Errors (in ms) for **actual** predictivity low vs. high (*Exp. 2*).

|  |  | actual predictivity low | | |  | actual predictivity high | | |
| --- | --- | --- | --- | --- | --- | --- | --- | --- |
|  |  | Gaze top | Gaze central | Gaze bottom |  | Gaze top | Gaze central | Gaze bottom |
|  |  |  |  |  |  |  |  |  |
| Target top | valid | 361 (17) | 364 (20) | 369 (19) |  | 330 (17) | 366 (21) | 403 (13) |
|  | invalid | 373 (19) | 374 (18) | 375 (22) |  | 381 (16) | 402 (23) | 410 (22) |
| Target central | valid | 348 (18) | 342 (18) | 344 (17) |  | 345 (21) | 322 (16) | 358 (21) |
|  | invalid | 363 (18) | 355 (18) | 359 (17) |  | 379 (15) | 400 (24) | 388 (19) |
| Target bottom | valid | 357 (18) | 351 (17) | 344 (17) |  | 370 (22) | 363 (23) | 319 (15) |
|  | invalid | 362 (18) | 364 (18) | 364 (18) |  | 389 (18) | 397 (22) | 387 (20) |
