# Supplementary material for: What We Observe Is Biased by What Other People Tell Us: Beliefs about the Reliability of Gaze Behavior Modulate Attentional Orienting to Gaze Cues
Source: PLoS One. 2014 Apr 10;9(4):e94529. doi: 10.1371/journal.pone.0094529 (PMC3983279; doi:10.1371/journal.pone.0094529)
Supplement: Table S5 — F-values and p-values for the four-way ANOVA on RTs with the factors (i) validity, (ii) gaze position, (iii) target position, and (iv) actual predictivity ( Exp. 2 ). (DOC) [file pone.0094529.s005.doc]

Table S5. F-values and p-values for the ANOVA on RTs with the factors: validity, gaze position, target position, and actual predictivity (*Exp.2*).

|  |  |  | *F-*value | *p-*value | effect size |
| --- | --- | --- | --- | --- | --- |
|  |  |  |  |  |  |
| validity |  |  | *F*(1,11)= 14.283 | *p=*.003 | η_P_^2^= .192 |
| target position |  |  | *F*(2,22)= 45.575 | *p*< .001 | η_P_^2^= .806 |
| gaze position |  |  | *F*(2,22)= 1.964 | *p*= .164 | η_P_^2^= .151 |
| predictivity |  |  | *F*(1,11)= 2.610 | *p*= .134 | η_P_^2^= .192 |
| validity x target position |  |  | *F*(2,22)= 9.018 | *p=* .001 | η_P_^2^= .451 |
| validity x gaze position |  |  | *F*(2,22)= 1.937 | *p*= .168 | η_P_^2^= .150 |
| validity x predictivity |  |  | *F*(1,11)= 10.765 | *p*= .007 | η_P_^2^= .495 |
| gaze position x target position |  |  | *F*(4,44)= 15.948 | *p*< .001 | η_P_^2^= .592 |
| gaze position x predictivity |  |  | *F*(2,22)= 3.022 | *p*= .069 | η_P_^2^= .216 |
| target position x predictivity |  |  | *F*(2,22)= 12.946 | *p*= .104 | η_P_^2^= .167 |
| validity x gaze pos x target pos |  |  | *F*(4,44)= 5.638 | *p*= .001 | η_P_^2^= .339 |
| validity x gaze pos x predictivity |  |  | *F*(2,22)= 2.203 | *p*= .134 | η_P_^2^= .167 |
| validity x target pos x predictivity |  |  | *F*(2,22)= 2.320 | *p*= .122 | η_P_^2^= .174 |
| gaze pos x target pos x predictivity |  |  | *F*(4,44)= 15.704 | *p*< .001 | η_P_^2^= .588 |
| validity x gaze pos x target pos x predictivity |  |  | *F*(4,44)= 5.018 | *p*= .002 | η_P_^2^= .313 |
|  |  |  |  |  |  |
