# Supplementary material for: What We Observe Is Biased by What Other People Tell Us: Beliefs about the Reliability of Gaze Behavior Modulate Attentional Orienting to Gaze Cues
Source: PLoS One. 2014 Apr 10;9(4):e94529. doi: 10.1371/journal.pone.0094529 (PMC3983279; doi:10.1371/journal.pone.0094529)
Supplement: Table S6 — F-values and p-values for the three-way ANOVA on gaze-cueing effects with the factors (i) gaze position, (ii) target position, and (iii) actual predictivity ( Exp. 2 ). (DOC) [file pone.0094529.s006.doc]

Table S6. F-values and p-values for the ANOVA on gaze-cueing effects with the factors: gaze position, target position, and actual predictivity (*Exp.2*).

|  |  |  | *F-*value | *p-*value | effect size |
| --- | --- | --- | --- | --- | --- |
|  |  |  |  |  |  |
| target position |  |  | *F*(2,22)= 9.018 | *p*= .001 | η_P_^2^= .451 |
| gaze position |  |  | *F*(2,22)= 1.937 | *p*= .168 | η_P_^2^= .150 |
| predictivity |  |  | *F*(1,11)= 10.765 | *p*= .007 | η_P_^2^= .495 |
|  |  |  |  |  |  |
| gaze position x target position |  |  | *F*(4,44)= 5.638 | *p*= .001 | η_P_^2^= .339 |
| gaze position x predictivity |  |  | *F*(2,22)= 2.203 | *p*= .134 | η_P_^2^= .167 |
| target position x predictivity |  |  | *F*(2,22)= 2.320 | *p*= .122 | η_P_^2^= .174 |
|  |  |  |  |  |  |
| gaze pos x target pos x predictivity |  |  | *F*(4,44)= 5.018 | *p*= .002 | η_P_^2^= .313 |
|  |  |  |  |  |  |
