# Supplementary material for: What We Observe Is Biased by What Other People Tell Us: Beliefs about the Reliability of Gaze Behavior Modulate Attentional Orienting to Gaze Cues
Source: PLoS One. 2014 Apr 10;9(4):e94529. doi: 10.1371/journal.pone.0094529 (PMC3983279; doi:10.1371/journal.pone.0094529)
Supplement: Table S7 — Mean Response Times and Standard Errors (in ms) for actual predictivity low/believed predictivity high vs. actual predictivity high/believed predictivity low (Exp.3). (DOC) [file pone.0094529.s007.doc]

**Table S7.** Mean Response Times and Standard Errors (in ms) for **actual** predictivity low/**believed** predictivity high vs. **actual** predictivity high/**believed** predictivity low (*Exp.3*).

|  |  | actual pred. low/ believed pred. high | | |  | actual pred. high/ believed pred. low | | |
| --- | --- | --- | --- | --- | --- | --- | --- | --- |
|  |  | Gaze top | Gaze central | Gaze bottom |  | Gaze top | Gaze central | Gaze bottom |
|  |  |  |  |  |  |  |  |  |
| Target top | valid | 280 (13) | 321 (20) | 338 (19) |  | 315 (14) | 330 (14) | 339 (13) |
|  | invalid | 358 (11) | 361 (13) | 381 (15) |  | 345 (12) | 347 (13) | 345 (12) |
| Target central | valid | 292 (15) | 275 (12) | 301 (18) |  | 311 (13) | 306 (14) | 312 (11) |
|  | invalid | 357 (12) | 358 (10) | 363 (11) |  | 333 (13) | 331 (10) | 333 (10) |
| Target bottom | valid | 337 (22) | 312 (19) | 273 (13) |  | 337 (13) | 332 (14) | 309 (12) |
|  | invalid | 360 (12) | 361 (11) | 349 (10) |  | 342 (12) | 340 (11) | 342 (10) |
