# Supplementary material for: What We Observe Is Biased by What Other People Tell Us: Beliefs about the Reliability of Gaze Behavior Modulate Attentional Orienting to Gaze Cues
Source: PLoS One. 2014 Apr 10;9(4):e94529. doi: 10.1371/journal.pone.0094529 (PMC3983279; doi:10.1371/journal.pone.0094529)
Supplement: Table S8 — F-values and p-values for the four-way ANOVA on RTs with the factors (i) validity, (ii) gaze position, (iii) target position, and (iv) actual predictivity. (DOC) [file pone.0094529.s008.doc]

**Table S8.**  F-values and p-values for the four-way ANOVA on **RTs** with the factors: validity, gaze position, target position, and **actual** predictivity (*Exp.3*).

|  |  |  | *F-*value | *p-*value | effect size |
| --- | --- | --- | --- | --- | --- |
|  |  |  |  |  |  |
| validity |  |  | *F*(1,11)= 59.829 | *p<*.001 | η_P_^2^= .845 |
| target position |  |  | *F*(2,22)= 16.975 | *p*< .001 | η_P_^2^= .607 |
| gaze position |  |  | *F*(2,22)= 1.261 | *p*= .303 | η_P_^2^= .103 |
| predictivity |  |  | *F*(1,11)= .074 | *p*= .791 | η_P_^2^= .007 |
| validity x target position |  |  | *F*(2,22)= 4.086 | *p=* .031 | η_P_^2^= .271 |
| validity x gaze position |  |  | *F*(2,22)= .069 | *p*= .934 | η_P_^2^= .006 |
| validity x predictivity |  |  | *F*(1,11)= 29.578 | *p*< .001 | η_P_^2^= .729 |
| gaze position x target position |  |  | *F*(4,44)= 15.421 | *p*< .001 | η_P_^2^= .584 |
| gaze position x predictivity |  |  | *F*(2,22)= .590 | *p*= .563 | η_P_^2^= .051 |
| target position x predictivity |  |  | *F*(2,22)= .979 | *p*= .391 | η_P_^2^= .082 |
| validity x gaze pos x target pos |  |  | *F*(4,44)= 10.847 | *p*< .001 | η_P_^2^= .496 |
| validity x gaze pos x predictivity |  |  | *F*(2,22)= .398 | *p*= .676 | η_P_^2^= .035 |
| validity x target pos x predictivity |  |  | *F*(2,22)= 1.873 | *p*= .177 | η_P_^2^= .145 |
| gaze pos x target pos x predictivity |  |  | *F*(4,44)= 4.794 | *p*= .003 | η_P_^2^= .304 |
| validity x gaze pos x target pos x predictivity |  |  | *F*(4,44)= 1.866 | *p*= .133 | η_P_^2^= .145 |
|  |  |  |  |  |  |
