# Supplementary material for: What We Observe Is Biased by What Other People Tell Us: Beliefs about the Reliability of Gaze Behavior Modulate Attentional Orienting to Gaze Cues
Source: PLoS One. 2014 Apr 10;9(4):e94529. doi: 10.1371/journal.pone.0094529 (PMC3983279; doi:10.1371/journal.pone.0094529)
Supplement: Table S9 — F-values and p-values for the four-way ANOVA on gaze-cueing effects with the factors (i) gaze position, (ii) target position, (iii) actual predictivity, and (iv) experiment (instructed predictivity, Exp. 3 ). (DOC) [file pone.0094529.s009.doc]

**Table S9.**  F-values and p-values for the four-way ANOVA on **gaze-cueing effects** with the factors: gaze position, target position, **actual** predictivity and experiment (**instructed** predictivity, *Exp.3*).

|  |  |  | *F-*value | *p-*value | effect size |
| --- | --- | --- | --- | --- | --- |
|  |  |  |  |  |  |
| actual predictivity |  |  | *F*(1,22)= 64.975 | *p*< .001 | η_P_^2^= .747 |
| target position |  |  | *F*(2,44)= 7.581 | *p=* .001 | η_P_^2^= .256 |
| gaze position |  |  | *F*(2,44)= .092 | *p*= .913 | η_P_^2^= .004 |
| experiment |  |  | *F*(1,22)= .222 | *p*= .642 | η_P_^2^= .010 |
|  |  |  |  |  |  |
| actual pred x experiment |  |  | *F*(1,22)= 1.146 | *p=* .296 | η_P_^2^= .049 |
| actual pred x gaze position |  |  | *F*(2,44)= .047 | *p*= .954 | η_P_^2^= .002 |
| actual pred x target position |  |  | *F*(2,44)= 3.715 | *p=* .032 | η_P_^2^= .144 |
| experiment x gaze position |  |  | *F*(2,44)= .612 | *p=* .547 | η_P_^2^= .027 |
| experiment x target position |  |  | *F*(2,44)= 1.278 | *p*= .289 | η_P_^2^= .055 |
| gaze position x target position |  |  | *F*(4,88)= 29.955 | *p<* .001 | η_P_^2^= .577 |
|  |  |  |  |  |  |
| actual pred x gaze pos x target pos |  |  | *F*(4,88)= 15.130 | *p*< .001 | η_P_^2^= .407 |
| experiment x gaze pos x target pos |  |  | *F*(4,88)= .634 | *p*= .639 | η_P_^2^= .028 |
| actual pred x experiment x gaze pos |  |  | *F*(2,44)= .766 | *p*= .471 | η_P_^2^= .034 |
| actual pred x experiment x target pos |  |  | *F*(2,44)= .097 | *p=* .908 | η_P_^2^= .004 |
|  |  |  |  |  |  |
| actual pred x experiment x target pos x gaze pos |  |  | *F*(4,88)= 5.419 | *p=* .001 | η_P_^2^= .198 |
|  |  |  |  |  |  |
