# Supplementary material for: What We Observe Is Biased by What Other People Tell Us: Beliefs about the Reliability of Gaze Behavior Modulate Attentional Orienting to Gaze Cues
Source: PLoS One. 2014 Apr 10;9(4):e94529. doi: 10.1371/journal.pone.0094529 (PMC3983279; doi:10.1371/journal.pone.0094529)
Supplement: Table S10 — F-values and p-values for the three-way ANOVA on gaze-cueing effects with the factors (i) gaze position, (ii) target position, and (iii) experiment (instructed predictivity, Exp. 3 ) for actual predictivity high vs. low. (DOC) [file pone.0094529.s010.doc]

**Table S10.**  F-values and p-values for the three-way ANOVA on **gaze-cueing effects** with the factors: gaze position, target position, and experiment (**instructed** predictivity, *Exp.3*) for actual predictivity high vs. low.

|  |  | actual predictivity high | | |  | actual predictivity low | | |
| --- | --- | --- | --- | --- | --- | --- | --- | --- |
|  |  | *F-*value | *p-*value | effect size |  | *F-*value | *p-*value | effect size |
|  |  |  |  |  |  |  |  |  |
| experiment |  | *F*(1,22)= .080 | *p=* .780 | η_P_^2^= .004 |  | *F*(1,22)= 7.051 | *p=* .014 | η_P_^2^= .243 |
| target position |  | *F*(2,44)= 6.291 | *p=* .004 | η_P_^2^= .222 |  | *F*(2,44)= 2.968 | *p=* .062 | η_P_^2^= .119 |
| gaze position |  | *F*(2,44)= .024 | *p=* .976 | η_P_^2^= .001 |  | *F*(2,44)= .401 | *p=* .672 | η_P_^2^= .018 |
|  |  |  |  |  |  |  |  |  |
| experiment x target position |  | *F*(2,44)= .595 | *p=* .556 | η_P_^2^= .026 |  | *F*(2,44)= 2.153 | *p=* .128 | η_P_^2^= .089 |
| experiment x gaze position |  | *F*(2,44)= .469 | *p=* .629 | η_P_^2^= .021 |  | *F*(2,44)= 2.152 | *p=* .128 | η_P_^2^= .089 |
| target position x gaze position |  | *F*(4,88)= 24.200 | *p*< .001 | η_P_^2^= .524 |  | *F*(4,88)= 11.728 | *p<* .001 | η_P_^2^= .348 |
|  |  |  |  |  |  |  |  |  |
| experiment x gaze pos x target pos |  | *F*(4,88)= 2.583 | *p=* .043 | η_P_^2^= .105 |  | *F*(4,88)= 5.649 | *p<* .001 | η_P_^2^= .204 |
|  |  |  |  |  |  |  |  |  |
